# Silencing TLR4 using an ultrasound-targeted microbubble destruction-based shRNA system reduces ischemia-induced seizures in hyperglycemic rats

**DOI:** 10.1515/biol-2022-0526

**Published:** 2022-12-27

**Authors:** Jia Chen, Fami Huang, Xiaobo Fang, Siying Li, Yanling Liang

**Affiliations:** Department of Neurology, The Third Affiliated Hospital of Guangzhou Medical University, 63 Duobao Road, Liwan District, Guangzhou, 510150, China; Department of Intensive Care Unit, The Sixth Affiliated Hospital of Guangzhou Medical University, Qingyuan, 511500, China; Key Laboratory for Major Obstetric Diseases of Guangdong Province, Guangzhou, 510150, China

**Keywords:** UTMD, TLR4, post-ischemic, seizures, hyperglycemia

## Abstract

The toll-like receptor 4 (TLR4) pathway is involved in seizures. We investigated whether ultrasound-targeted microbubble destruction (UTMD)-mediated delivery of short hairpin RNA (shRNA) targeting the *TLR4* gene (shRNA-TLR4) can reduce ischemia-induced seizures in rats with hyperglycemia. A total of 100 male Wistar rats were randomly assigned to five groups: (1) Sham; (2) normal saline (NS); (3) shRNA-TLR4, where rats were injected with shRNA-TLR4; (4) shRNA-TLR4 + US, where rats were injected with shRNA-TLR4 followed by ultrasound (US) irradiation; and (5) shRNA-TLR4 + microbubbles (MBs) + US, where rats were injected with shRNA-TLR4 mixed with MBs followed by US irradiation. Western blot and immunohistochemical staining were used to measure TLR4-positive cells. Half of the rats in the NS group developed tonic-clonic seizures, and TLR4 expression in the CA3 region of the hippocampus was increased in these rats. In addition, the NS group showed an increased number of TLR4-positive cells compared with the Sham group, while there was a decreased number of TLR4-positive cells in the shRNA, shRNA + US, and shRNA + MBs + US groups. Our findings indicate that the TLR4 pathway is involved in the pathogenesis of ischemia-induced seizures in hyperglycemic rats and that UTMD technology may be a promising strategy to treat brain diseases.

## 1 Introduction

Epilepsy is a neurological disorder involving unprovoked, recurring seizures [1]. These seizures often lead to brain injury and persistent psychogenic and neurologic disorders, which significantly burden patients, their families, and society [2]. Seizures may occur during the acute phase of a stroke [3]. Previous studies have shown that hyperglycemic rats have a markedly increased incidence of post-ischemic attacks, and hyperglycemia is considered a major risk factor that increases the frequency of seizures and the severity of epilepsy [4,5,6,7,8]. In approximately 30% of affected individuals, epilepsy cannot be treated pharmacologically [9], and surgical treatment is only suitable for a small proportion of patients. Thus, a better understanding of the pathophysiology of seizures is essential for developing novel therapeutic approaches for epileptic seizures.

Inflammatory factors and signaling pathways are highly involved in the pathogenesis of epileptic seizures and epilepsy [10,11,12]. Previous evidence has revealed that the toll-like receptor (TLR) 4 pathway is involved in seizures following global ischemia with hyperglycemia [7], which provided insights into the pathogenesis of seizures resulting from hyperglycemic ischemia. TLRs are expressed in mammalian cells and play critical roles in activating and regulating immune/inflammatory responses [13]. Endogenous TLR ligands are a group of molecules released from tissues after injury or cellular stress and are therefore termed damage-associated molecular patterns. Excessive release of these molecules, such as heat shock protein and high-mobility group box-1, may trigger inflammation. TLRs recognize molecules of microbial origin, called pathogen-associated molecular patterns (e.g., lipopolysaccharide), and induce inflammation by activating the transcription of genes encoding inflammatory factors (e.g., interleukin (IL)-1β) [14,15]. TLR4 was the first recognized member of the TLR family and is widely expressed in primary sensory neurons and glial cells. It is a vital transmembrane pattern-recognition receptor of the innate immune system and is involved in different pathological processes, such as sepsis, cardiac diseases, and ischemia/reperfusion injury [16,17]. TLR4 is also implicated in acute symptomatic seizures and contributes to an increased risk of chronic epilepsy [19]. TLR4 knockout and TLR4 antagonists (e.g., high-mobility group box-1) have been shown to reduce the occurrence of seizures [12,18,19].

Ultrasound-targeted microbubble destruction (UTMD) technology is a promising *in vitro* and *in vivo* gene transfection method [20]. UTMD creates transient pores in cell membranes and increases cell membrane permeability to small molecules [21,22]. RNA interference (RNAi) is a biological process that induces post-transcriptional sequence-specific gene silencing. RNAi-mediated gene silencing effectively regulates tumor cells, abnormal tissues, and protein mutations. It has been widely applied in gene function analysis and gene therapy [23]. The primary RNA types involved in RNAi include double-stranded vector-based short hairpin RNA (shRNA), small interfering RNA, and microRNA. The use of shRNA enables more effective and stable gene silencing compared to other types of RNAs [24,25]. The UTMD-based shRNA delivery system has been shown to efficiently knock down survival genes *in vivo*, thereby inducing cell apoptosis and inhibiting proliferation [26,27,28].

The present study aimed to investigate the efficiency of UTMD-based shRNA delivery for silencing *TLR4* gene expression and whether *TLR4* silencing can prevent ischemia-induced seizures in hyperglycemic rats.

## 2 Materials and methods

### 2.1 Construction of shRNA plasmid targeting TLR4 (pshRNA-TLR4)

The pshRNA-TLR4 was constructed by JiMa Pharmaceutical Technology (Shanghai, China). The shRNA sequence was 5′-TTAAGAAGCTATAGCTTCACC-3′.

### 2.2 Preparation of SonoVue microbubbles (MBs) mixed with pshRNA-TLR4

SonoVue MBs were suspended in 0.9% normal saline (NS) to a concentration of 1 × 10^8^/mL. A volume of 100 µL suspension was mixed with 100 μL pshRNA-TLR4 (2 μg/μL) at 4°C for 30 min to obtain the SonoVue MBs/pshRNA-TLR4 mixture [29,30].

### 2.3 Animals

One hundred adult male Wistar rats (12–14 weeks old, 200–250 g) were obtained from the Experimental Animal Center at Southern Medical University (Guangzhou, China) and housed in an environment with a 12:12 h light/dark cycle. All rats were provided with sufficient water and food during the study. Rats were randomly assigned to five groups (*n* = 20 per group): (1) Sham; (2) NS, where rats were administered with 0.9% NS (0.1 µL/g) through an injection into the right lateral ventricle. During the surgical procedure, animals were secured in a stereotaxic frame (Stoelting, Wood Dale, IL, USA) with the incisor bar at −3.3 mm. A small hole was made in the skull at the height of the right lateral ventricle located based on stereotactic coordinates in relation to bregma (AP −1.0 mm, LA −1.5 mm, DV −3.8 mm); (3) pshRNA, where rats were injected with pshRNA-TLR4 (0.1 µL/g) in the right lateral ventricle; (4) pshRNA + ultrasound (US), where rats were injected with pshRNA-TLR4 (0.1 µL/g) in the right lateral ventricle, followed by US irradiation; and (5) pshRNA + MBs + US, where rats were injected with a mixture of SonoVue MBs with 20 µL [31] of pshRNA-TLR4 in the right lateral ventricles, followed by US irradiation.

Rats were anesthetized by an intraperitoneal (i.p.) injection of 3% pentobarbital sodium (1 mL/kg) and then injected with 0.9% NS, pshRNA-TLR4, or SonoVue MBs mixed with pshRNA-TLR4 in the right lateral ventricle under stereotactic guidance. Subsequently, the injected region was exposed to US irradiation for 5 min using a US-emitting transducer (15 mm in diameter; Rimed, Israel). The parameters of US irradiation were as follows: frequency, 2 MHz [32,33]; average intensity, 1.5 W/cm^2^; and depth, 38 mm. After ultrasound irradiation, the rats were placed in a dry and warm place, awaiting resuscitation from anesthesia.


**Ethical approval:** The research related to animal use has been complied with all the relevant national regulations and institutional policies for the care and use of animals. This study was approved by the Animal Ethics Committee of Guangzhou Medical Laboratory Animal Center and performed following the NIH Guide for the Care and Use of Laboratory Animals. The Laboratory Animal Care and Use Committee of the Guangzhou Medical University reviewed and approved all experimental protocols involving animals. The ethical code was SCXK2011-0015. Clinical signs, such as limb weakness and movement, were examined daily by an investigator blinded to the study. The same investigator performed subsequent tissue sample analyses.

### 2.4 Induction of transient global cerebral ischemia

All rats except those in the Sham group were anesthetized with 1 mL/kg of 3% pentobarbital sodium (i.p.). Transient global cerebral ischemia was induced following the modified four-vessel occlusion method [34]. A silicone tube was used as an occlusion device and placed loosely around each common carotid artery. On Day 1, the targeted rat was placed in a stereotaxic frame, and the vertebral arteries were electrocauterized. On Day 2, both common carotid arteries were occluded using the silicone tube while the target animal was awake. Complete ischemia was defined as loss of the righting reflex and unresponsiveness within 1 min. Rats without the aforementioned symptoms were regarded as unsuccessful in modeling and were excluded from the group. After 15 min, rats that met the criteria of complete ischemia were reperfused by releasing the occlusion of both carotid arteries. Rats were then dried and placed under a heat lamp until they recovered from the ischemia.

### 2.5 Induction of hyperglycemia

Hyperglycemia (>200 mg/dL) was induced in all rats except for those in the Sham group by a single injection of glucose (3 g/kg, i.p.) 15 min before the induction of ischemia. Rats with blood glucose <200 mg/mL were excluded from the group.

### 2.6 Immunohistochemical staining

**Figure 1:**
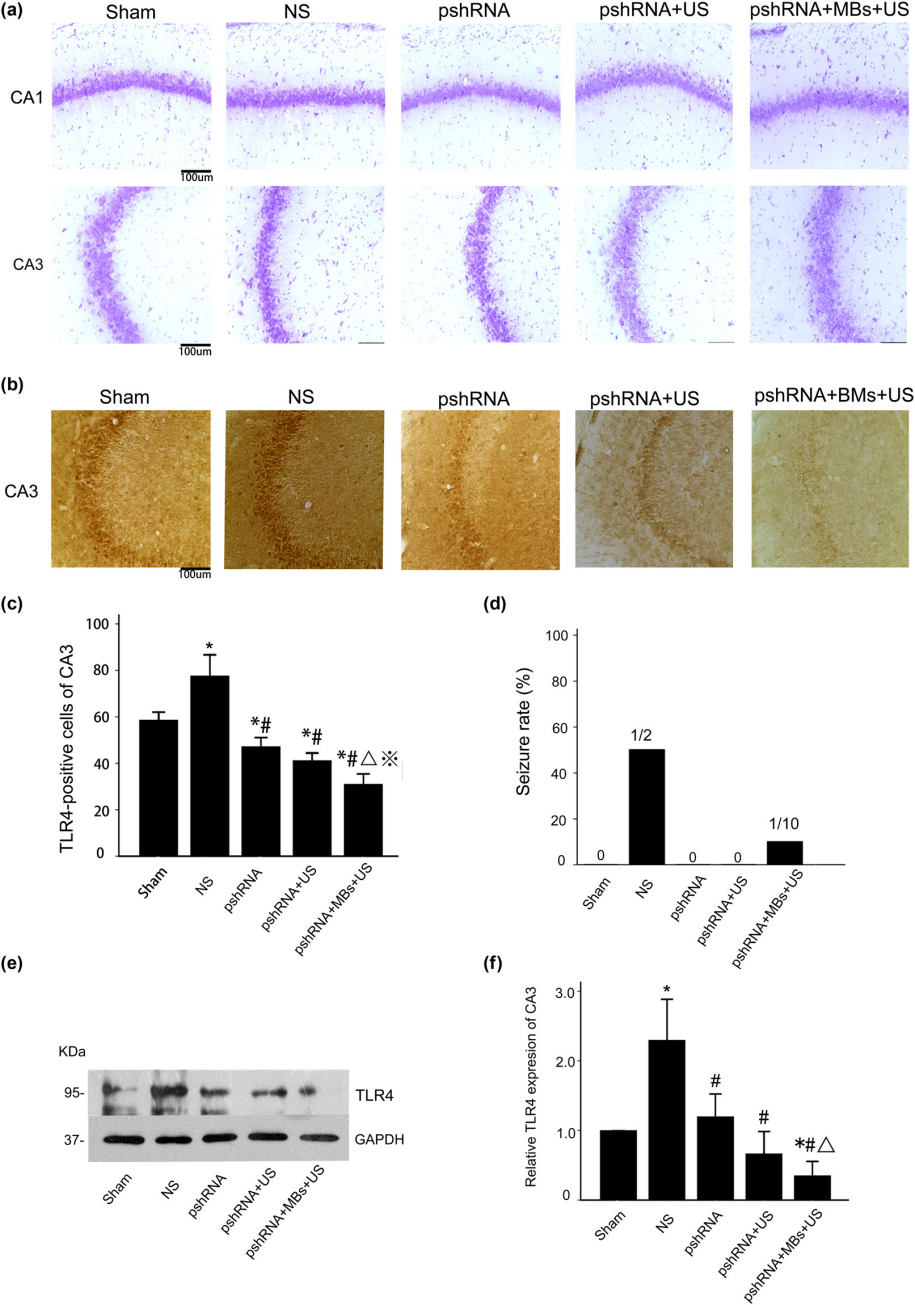
The effect of *TLR4* silencing in rats with hyperglycemic ischemia: (a) Nissl staining showed no cell death in any group at 6 h post-ischemia. (b) TLR4 expression in the CA3 subregion of hyperglycemic rats (the NS group) that developed tonic-clonic seizure after ischemia was significantly increased compared with the Sham group. (c) The number of TLR4-positive cells in the pshRNA, pshRNA + US, and pshRNA + MBs + US groups after ischemia significantly decreased compared with the NS group. *p < 0.05 vs the Sham group; #*p* < 0.05 vs the NS group; ^Δ^ *p* < 0.01 vs the pshRNA group; ※ *p* < 0.05 vs the pshRNA+US group. (d) More rats in the NS group developed tonic-clonic seizures after ischemia compared with the pshRN+MBs+US group (50 vs 10%). (e and f) Western blot analysis of TLR4 expression in the CA3 region of all groups. Protein expression of TLR4 in NS-treated rats that developed tonic-clonic seizure after ischemia significantly increased compared with the Sham group. TLR4 was significantly upregulated in the pshRNA, pshRNA + US, and pshRNA + MB + U S groups. GAPDH was used as a loading control. Data are presented as mean ± SD from five independent experiments. One-way ANOVA was used to analyze statistical differences. *p < 0.05 vs the Sham group; #*p* < 0.05 vs the NS group; ^Δ^ *p* < 0.01 vs the pshRNA group.

Rats (*n* = 5 per group) were anesthetized and perfused with 0.9% NS for 5 min, followed by 4% paraformaldehyde diluted in phosphate-buffered saline (PBS) for 30 min through the ascending aorta. Each rat brain was then harvested and fixed in 4% paraformaldehyde at 4°C overnight. Coronal sections (30 μm) containing the hippocampus were cut using a vibratome (Leica, Germany). A proportion of the sections was used for Nissl staining. After three washes with PBS, the sections were incubated with 0.3% H_2_O_2_ diluted in PBS for 30 min to quench endogenous peroxidase activity. The sections were then blocked and permeabilized in a permeabilization solution (5% bovine serum albumin in PBS) for 1 h at room temperature. Next, tissue sections were incubated with a rabbit polyclonal anti-TLR4 antibody (1:200; Abcam, USA) at 4°C overnight. After seven PBS washes (5 min per wash), the sections were incubated with a biotinylated goat anti-rabbit IgG at room temperature for 2 h and then with the avidin–biotin–peroxidase complex for 30 min. After five PBS washes (5 min per wash), the sections were processed with the ABC staining kit (Fuzhou Maixin Biotechnology Development, China) and visualized using 0.05% diaminobenzidine tetrahydrochloride. All samples were stained together in each session, and all sections were exposed to diaminobenzidine tetrahydrochloride at the same time. Tissue slices were then mounted onto slides, air-dried, dehydrated, and incubated in xylene. Images were captured using a digital camera at 40× and 200× magnifications. ImageJ software was used to quantify TLR4-positive cells in the CA3 subregion of the hippocampus. Representative sections were selected from each brain sample. In each slide, one field in the CA3 region was captured using a rectangular frame (1,500 μm × 8,500 μm, 200× magnification, Figure 1a) with a digital camera.

### 2.7 Western blot analysis

Brain tissue samples were prepared for Western blot analysis as previously described [35]. Briefly, rats were anesthetized with an overdose of pentobarbital sodium (50 mg/kg) and decapitated. The brains were quickly removed and immersed in ice-cold PBS containing 2 mmol/L KH_2_PO_4_, 10 mmol/L Na_2_HPO_4_, 137 mmol/L NaCl, and 2.7 mmol/L KCl (pH = 7.4). The hippocampal tissues were cut into four pieces. The CA3 subregion of the hippocampus was quickly micro-dissected under a surgical microscope (Leica, Dmirb, Germany) and then frozen in liquid nitrogen. The tissues were incubated with ice-chilled radioimmunoprecipitation assay buffer (Biyuntian, China) supplemented with a protease inhibitor cocktail (Biyuntian) on ice for 30 min. The samples were centrifuged at 12,000×*g* at 4°C for 10 min, and the supernatant was collected. The protein concentration was measured using the BCA protein assay (Bio-Rad, USA). 2 × SDS gel-loading buffer (Sigma, USA) was added to the samples and boiled. Proteins (100 μg) were separated using 8% SDS–PAGE and transferred to 0.45 mm polyvinylidene difluoride membranes (Bio-Rad) in transfer buffer. The membranes were rinsed with distilled water, blocked with 5% dry milk (Bio-Rad) in Tris-buffered saline 0.1% Tween 20 (TBST) at room temperature for 60 min, and then incubated with rabbit polyclonal anti-TLR4 antibody (1:1,000, Abcam) overnight at 4°C. The membranes were washed with TBST seven times (7 min per wash) and incubated with an anti-rabbit HRP-conjugated secondary antibody (1:10,000, Abcam) at room temperature for 2 h. The membranes were rinsed in TBST eight times. The signals were detected using enhanced chemiluminescence (Bio-Rad) and visualized using X-ray (Keda, China). The band density was analyzed using Quantity One software. The expression of TLR4 was normalized to that of GAPDH. All values are shown as the ratio relative to the control group.

### 2.8 Statistical analysis

Data are presented as the mean ± standard deviation (SD). SPSS 16.0 software was used for statistical analysis. Differences among the experimental groups were analyzed using the two-tailed student *t*-test, ANOVA, or the Mann–Whitney *U* nonparametric test as implemented in SPSS 16.0 (SPSS Inc., Chicago, IL). Tukey’s or Tamhane T2 *post-hoc* test was used for group-wise comparisons to determine significant differences. A value of *p* < 0.05 was considered statistically significant.

## 3 Results

### 3.1 Seizure rate and brain damage

We use the Racine scale as the criterion for epilepsy. The seizure was recorded as grade V behavioral manifestations (generalized tonic-clonic activity with loss of postural tone, often resulting in death and wild jumping). Half of the rats in the NS group developed tonic-clonic seizures within 3 h after the induction of ischemia. These rats died of status epilepticus within 2 h after the seizure onset. Rats in the pshRNA and pshRNA + US groups did not develop seizures at 0–6 h after ischemia. Two rats (2/20, 10%) in the pshRNA + MBs + US group developed seizures at 3–6 h after ischemia (Figure 1d).

At 6 h after ischemia, damage in the hippocampus was assessed using Nissl staining. No cell death was observed in any group (Figure 1a), suggesting that the expression level of TLR4 did not affect cell death in the hippocampus.

### 3.2 Expression of TLR4 in the CA3 subregion of the hippocampus

Immunohistochemical staining was used to detect TLR4-positive cells in the CA3 subregion of the hippocampus. As shown in Figure 1b and c and Table 1 and Table S1, rats that developed tonic-clonic seizures after ischemia showed significantly more TLR4-positive cells than the Sham group (77.6 ± 3.20 vs 58.6 ± 1.24, *p* < 0.05, *n* = 5 per group). The number of TLR4-positive cells in the pshRNA (47.2 ± 1.39), pshRNA + US (41.2 ± 1.15), and pshRNA + MBs + US (31.0 ± 1.58) groups after ischemia decreased compared with the number of positive cells in the NS group that developed seizures (all *p* < 0.05, *n* = 5 per group). In addition, there were fewer TLR4-positive cells in the pshRNA + MBs + US group compared to the pshRNA and pshRNA + US groups (*p* < 0.05).

**Table 1:** Comparison of Immunohistochemical staining data between each group (*x̅* ± *s*)

Groups	TLR4-positive cells of CA3	*F*
sham	58.600 ± 1.248	88.592
NS	77.600 ± 3.264^*#^	
pshRNA	47.200 ± 1.392^*#^	
pshRNA + US	41.200 ± 1.157^*#^	
pshRNA + MBs + US	31.000 ± 1.581^*Δ※^	

*F* value = 88.592. ^*^ *p* < 0.05 vs the Sham group; ^#^ *p* < 0.05 vs the NS group; ^Δ^ *p* < 0.01 vs the pshRNA group; ^※^ *p* < 0.05 vs the pshRNA + US group.

*p*-values for pairwise comparisons between groups are shown in Table S1.

The CA3 hippocampal region is one of the most susceptible brain regions to seizures [36]. Protein expression of TLR4 (95 kDa) in the CA3 region was measured using Western blot analysis. The NS rats that developed tonic-clonic seizures showed significantly upregulated TLR4 expression compared with the Sham group (2.30 ± 0.47 vs 1.00 ± 0.00, *p* < 0.05, *n* = 5 per group). The relative expression of TLR4 in the pshRNA (1.20 ± 0.26), pshRNA + US (0.67 ± 0.26), and pshRNA + MBs + US (0.35 ± 0.17) groups after ischemia significantly decreased compared with the NS group (all *p* < 0.05, *n* = 5 per group). TLR4 expression in the pshRNA + MBs + US group was significantly downregulated compared with the pshRNA group (*p* < 0.05). No difference in TLR4 expression was observed between the pshRNA + US and pshRNA + MBs + US groups (Figure 1e and f, Table 2 and Table S2).

**Table 2:** Comparison of western blot data between each group (*x̅* ± *s*)

Groups	Relative TLR4 expression of CA3	*F*
sham	1.000 ± 0.001	35.690
NS	2.296 ± 0.211^*^	
pshRNA	1.198 ± 0.116^#^	
pshRNA + US	0.666 ± 0.115^#^	
pshRNA + MBs + US	0.349 ± 0.074^*#Δ^	

*F* value = 35.690. ^*^ *p* < 0.05 vs the Sham group; ^#^ *p* < 0.05 vs the NS group; ^Δ^ *p* < 0.01 vs the pshRNA group.

*p*-values for pairwise comparisons between groups are shown in Table S2.

## 4 Discussion

A better understanding of the mechanisms of seizure induction may contribute to developing advanced therapeutic strategies for this neurological disorder. In this study, we reported that the TLR4 pathway was involved in the pathogenesis of ischemia-induced seizures in hyperglycemic rats.

Pre-ischemic hyperglycemia deteriorates brain function and aggravates neuronal damage after ischemia, ultimately causing post-ischemic seizures. Hyperglycemia also significantly increases the incidence of post-ischemic attacks [37]. TLRs are critical regulators of immune and inflammatory responses, which are highly involved in the pathogenesis of seizures [38]. In recent years, there has been increasing clinical and experimental support for the involvement of inflammatory and immune processes in the pathogenesis of seizures and epilepsy [10,39]. Inflammatory responses to stroke are associated with acute symptomatic seizures and a high risk of developing epilepsy [40,41].

Activation of the specific pro-inflammatory signaling TLR4 pathway is associated with seizure precipitation and recurrence in experimental models [42,43]. Among them, TLR4 participates in the progression of cerebral ischemia and reperfusion [44,45,46,47,48]. TLR4 is widely expressed in the nervous system (i.e., neurons, astrocytes, and microglia) and is implicated in various neurological disorders [36]. Hyperglycemia has been shown to upregulate TLR4, leading to excessive inflammatory reactions [49,50,51,52]. In this study, TLR4 was mainly upregulated in the CA3 region of hyperglycemic rats with ischemia-induced seizures, and this finding was not correlated with ischemic cell death. Half of the hyperglycemic rats in the NS group developed tonic-clonic seizures within 3 h after ischemia.

Hyperglycemia-induced upregulation of TLR4 is associated with increased NADPH oxidase activity via protein kinase C [53]. It has also been suggested that advanced glycation end-products increase the activity of TLRs [54]. Hyperglycemia has been reported to exacerbate ischemic brain damage in rats [55]. To determine whether neuronal damage contributed to seizures following ischemia in hyperglycemic rats, we performed Nissl staining to examine brain damage in each group. No cell death was observed in the hyperglycemic rats with ischemia-induced seizures. A possible reason for the lack of cell death could be that rats developed seizures approximately 6 h after reperfusion. Thus, morphological changes (*i.e.*, cell death) cannot be detected at this time. Neurons in the CA1 region begin to die 2–3 days after 10–15 min of global ischemia, and maximal cell death is observed 7 days post-ischemia [56]. In diabetic animals, neuronal death occurs 7–14 days after 10 min of ischemia [37]. No cell death is found at the early onset of seizures [7].

UTMD is a new drug delivery technology with several advantages in gene delivery [57], such as target-specificity and few adverse effects. UTMD has been applied in animal and *in vitro* studies [58,59]. Compared with naked plasmids, UTMD-mediated plasmids have significantly improved transfection efficiency [60,61]. Shen et al. [62] found that US-mediated pGL3 plasmids increased the delivery efficiency of a luciferase gene by 2- to 10-fold in mouse livers [63], while the use of UTMD-mediated plasmids resulted in an 85-fold increase in transfection efficiency. Deng et al. [64] found that UTMD combined with the NF-κB binding motif increased transfection efficiency by enhancing the cytoplasmic and nuclear intake of plasmids. shRNA is an effective tool for stable knockdown of gene expression. The UTMD-mediated shRNA knockdown of connective transforming growth factor inhibited the expression of the target gene and ameliorated progressive renal fibrosis [65,66].

Our previous study found that the expression level of TLR4 was associated with epileptic seizures in rats after transient hyperglycemia and global cerebral ischemia. The change in seizure rate, hyperglycemia, and the whole used TLR4 as the target to construct a short hairpin shRNA plasmid. The shRNA plasmid was injected into the lateral ventricle of the rat using UTMD. The target gene TLR4 was silenced, and the brain was observed. These studies confirmed the role of TLR4 and that UTMD can be used as an effective means of treating brain diseases.

This study evaluated the effect of UTMD transfer of shRNA against TLR4 in hyperglycemic rats with seizures. The shRNA-MBs complex circulated to the blood–brain barrier. TLR4 expression was significantly upregulated in the NS-treated rats at 15 min post-ischemia, consistent with a previous study showing that TLR4 is implicated in seizures following ischemia with hyperglycemia [7]. Compared with the Sham group, the pshRNA group showed reduced expression of TLR4. In addition, the pshRNA and pshRNA + US groups showed decreased expression of TLR4 in the CA3 region. However, no significant difference was found between the two groups. Rats treated with UTMD-mediated shRNA plasmid had the lowest expression of TLR4 among all groups. The pshRNA + MBs + US group also showed a decreased seizure rate compared with the NS group. Thus, these data demonstrate that UTMD-mediated shRNA plasmid efficiently silenced *TLR4* expression and reduced the incidence of seizures following ischemia in hyperglycemic rats.

## 5 Conclusions

In conclusion, silencing *TLR4* using UTMD-based shRNA reduced ischemia-induced seizures in hyperglycemic rats. Our study indicates that UTMD technology is an efficient and safe tool for gene silencing and may be used in gene therapy for brain diseases. Further studies are needed to determine the optimal concentration of plasmids and MBs, as well as the types of MBs and vectors, to enhance transfection efficiency.

## Supplementary Material

Supplementary Figure
