# Supplementary material for: Silencing TLR4 using an ultrasound-targeted microbubble destruction-based shRNA system reduces ischemia-induced seizures in hyperglycemic rats
Source: Open Life Sci. 2022 Dec 27;17(1):1689–97. doi: 10.1515/biol-2022-0526 (PMC9795576; doi:10.1515/biol-2022-0526)
Supplement: Supplementary Figure [file biol-2022-0526-sm.pdf]

Supplementary material

Table S1:

TLR4 positive cells of CA3 (Immunohistochemical staining analysis)						
**Groups**	**N**	**Mean value**	**standard deviation**	**standard error**	**95% confidence interval of the mean**	
					**lower limits**	**upper limits**
sham	5	58.600000000000000	2.792848008753788	1.248999599679679	55.132221175075350	62.067778824924650
NS	5	77.600000000000000	7.300684899377593	3.264965543462902	68.535002398212970	86.664997601787020
pshRNA	5	47.200000000000000	3.114482300479487	1.392838827718412	43.332859454451786	51.067140545548220
pshRNA + US	5	41.200000000000000	2.588435821108956	1.157583690279022	37.986032429268010	44.413967570732000
pshRNA + MBs + US	5	31.000000000000000	3.535533905932738	1.581138830084190	26.610054834574584	35.389945165425420
Total	25	51.120000000000000	16.756391815264603	3.351278363052921	44.203301406880420	58.036698593119574

Analysis of variance

1

	**Sum of squares**	**df**	**Mean squared**	***F***	**Significance**
Between groups	6378.640	4	1594.660	88.592	0.000
Within the group	360.000	20	18.000		
Total	6738.640	24			

Multiple comparisons

	**(I) 1**	**(J) 1**	**Average difference (I-J)**	**Standard error**	**Significance**	**95% Confidence interval**	
						**Lower limits**	**Upper limits**
LSD	sham	NS	−18.999999999999993*	2.683281572999748	0.000	−24.597227279999284	−13.402772720000701
		pshRNA	11.399999999999999*	2.683281572999748	0.000	5.802772720000706	16.997227279999290
		pshRNA + US	17.400000000000000*	2.683281572999748	0.000	11.802772720000707	22.997227279999290
		pshRNA + MBs + US	27.600000000000000*	2.683281572999748	0.000	22.002772720000710	33.197227279999300

(*Continued*)

Table S1: *Continued*

Multiple comparisons							
	(I) 1	(J) 1	Average difference (I-J)	Standard error	Significance	95% Confidence interval	
						Lower limits	Upper limits
	NS	sham	18.999999999999993*	2.683281572999748	0.000	13.402772720000701	24.597227279999284
		pshRNA	30.399999999999990*	2.683281572999748	0.000	24.802772720000700	35.997227279999290
		pshRNA + US	36.399999999999990*	2.683281572999748	0.000	30.802772720000700	41.997227279999290
		pshRNA + MBs + US	46.599999999999994*	2.683281572999748	0.000	41.002772720000700	52.197227279999290
	pshRNA	sham	−11.399999999999999*	2.683281572999748	0.000	−16.997227279999290	−5.802772720000706
		NS	−30.399999999999990*	2.683281572999748	0.000	−35.997227279999290	−24.802772720000700
		pshRNA + US	6.000000000000000*	2.683281572999748	0.037	.402772720000708	11.597227279999291
		pshRNA + MBs + US	16.200000000000003*	2.683281572999748	0.000	10.602772720000711	21.797227279999294
	pshRNA + US	sham	−17.400000000000000*	2.683281572999748	0.000	−22.997227279999290	−11.802772720000707
		NS	−36.399999999999990*	2.683281572999748	0.000	−41.997227279999290	−30.802772720000700
		pshRNA	−6.000000000000000*	2.683281572999748	0.037	−11.597227279999291	−0.402772720000708
		pshRNA + MBs + US	10.200000000000003*	2.683281572999748	0.001	4.602772720000711	15.797227279999294
	pshRNA + MBs + US	sham	−27.600000000000000*	2.683281572999748	0.000	−33.197227279999300	−22.002772720000710
		NS	−46.599999999999994*	2.683281572999748	0.000	−52.197227279999290	−41.002772720000700
		pshRNA	−16.200000000000003*	2.683281572999748	0.000	−21.797227279999294	−10.602772720000711
		pshRNA + US	−10.200000000000003*	2.683281572999748	0.001	−15.797227279999294	−4.602772720000711
Tamhane	sham	NS	−18.999999999999993*	3.495711658589707	0.026	−35.309977816444470	−2.690022183555509
		pshRNA	11.399999999999999*	1.870828693386970	0.003	4.231231348248572	18.568768651751427
		pshRNA + US	17.400000000000000*	1.702938636592640	0.000	10.888108041009780	23.911891958990218
		pshRNA + MBs + US	27.600000000000000*	2.014944167960989	0.000	19.766463653307138	35.433536346692870
	NS	sham	18.999999999999993*	3.495711658589707	0.026	2.690022183555509	35.309977816444470
		pshRNA	30.399999999999990*	3.549647869859770	0.002	14.305089644732195	46.494910355267790
		pshRNA + US	36.399999999999990*	3.464101615137755	0.001	19.934160895679410	52.865839104320570
		pshRNA + MBs + US	46.599999999999994*	3.627671429443411	0.000	30.720274559151516	62.479725440848470

(*Continued*)

Table S1: *Continued*

Multiple comparisons						
**(I) 1**	**(J) 1**	**Average difference (I-J)**	**Standard error**	**Significance**	**95% Confidence interval**	
					**Lower limits**	**Upper limits**
pshRNA	sham	−11.399999999999999*	1.870828693386970	0.003	−18.568768651751427	−4.231231348248572
	NS	−30.399999999999990*	3.549647869859770	0.002	−46.494910355267790	−14.305089644732195
	pshRNA + US	6.000000000000000	1.811077027627483	0.106	−.991949907294031	12.991949907294032
	pshRNA + MBs + US	16.200000000000003*	2.107130750570548	0.001	8.114276074445538	24.285723925554468
pshRNA + US	sham	−17.400000000000000*	1.702938636592640	0.000	−23.911891958990218	−10.888108041009780
	NS	−36.399999999999990*	3.464101615137755	0.001	−52.865839104320570	−19.934160895679410
	pshRNA	−6.000000000000000	1.811077027627483	0.106	−12.991949907294032	.991949907294031
	pshRNA + MBs + US	10.200000000000003*	1.959591794226542	0.011	2.480976160975501	17.919023839024504
pshRNA + MBs + US	sham	−27.600000000000000*	2.014944167960989	0.000	−35.433536346692870	−19.766463653307138
	NS	−46.599999999999994*	3.627671429443411	0.000	−62.479725440848470	−30.720274559151516
	pshRNA	−16.200000000000003*	2.107130750570548	0.001	−24.285723925554468	−8.114276074445538
	pshRNA + US	−10.200000000000003*	1.959591794226542	0.011	−17.919023839024504	−2.480976160975501

1

	**1**	**A Subset of alpha = 0.05**	
		**4**	**5**
Student-Newman-Keuls multiple comparisons [a]	pshRNA + MBs + US		
	pshRNA + US		
	pshRNA		
	sham	58.600000000000000	
	NS		77.600000000000000
	Significance	1.000	1.000

*Differences in means are significant at the 0.05 level. Will display the mean of the group in a homogeneous subset.[a]Use Harmonic Mean Sample Size = 5.000.

Table S2:

Relative TLR4 of express of CA3 (Western blot analysis)						
**Groups**	**N**	**Mean value**	**standard deviation**	**standard error**	**95% confidence interval of the mean**	
					**lower limits**	**upper limits**
sham	5	1.000000000000000	0.000000000000000	0.000000000000000	1.000000000000000	1.000000000000000
NS	5	2.296397011391140	0.473007859995542	0.211535545768347	1.709080180767269	2.883713842015012
pshRNA	5	1.198926826785087	0.260789340756725	0.116628538747879	0.875114091252168	1.522739562318007
pshRNA + US	5	0.666181748277171	0.257281118112274	0.115059613885239	0.346725046499550	0.985638450054791
pshRNA + MBs + US	5	0.349880449324186	0.166336271437537	0.074387842011638	0.143346689484747	0.556414209163624
Total		25	1.102277207155517	0.723200509488522	0.144640101897704	0.803754708895134
1.400799705415900						

Analysis of variance					
		1			
**Between groups**	**Sum of squares**	**df**	**Mean squared**	***F***	**b**
within the group	11.010	4	2.753	35.690	0.000
	1.542	20	0.077		
Total	12.552	24			

Multiple comparisons							
	**(I) 1**	**(J) 1**	**Average difference (I-J)**	**standard error**	**signifi-cance**	**95% Confidence interval**	
						**lower limits**	**upper limits**
LSD	sham	NS	−1.296397011391140*	0.175638001537875	0.000	−1.662771462549959	−0.930022560232322
		pshRNA	−0.198926826785087	0.175638001537875	0.271	−0.565301277943906	0.167447624373731
		pshRNA + US	0.333818251722829	0.175638001537875	0.072	−0.032556199435989	0.700192702881648
		pshRNA + MBs + US	0.650119550675814*	0.175638001537875	0.001	0.283745099516996	1.016494001834633

(*Continued*)

Table S2: *Continued*

	Multiple comparisons						
	(I) 1	(J) 1	Average difference (I-J)	standard error	signifi-cance	95% Confidence interval	
						lower limits	upper limits
	NS	sham	1.296397011391140*	0.175638001537875	0.000	0.930022560232322	1.662771462549959
		pshRNA	1.097470184606053*	0.175638001537875	0.000	0.731095733447235	1.463844635764871
		pshRNA + US	1.630215263113970*	0.175638001537875	0.000	1.263840811955151	1.996589714272788
		pshRNA + MBs + US	1.946516562066955*	0.175638001537875	0.000	1.580142110908136	2.312891013225773
	pshRNA	sham	0.198926826785087	0.175638001537875	0.271	−0.167447624373731	0.565301277943906
		NS	−1.097470184606053*	0.175638001537875	0.000	−1.463844635764871	−0.731095733447235
		pshRNA + US	0.532745078507917*	0.175638001537875	0.007	0.166370627349098	0.899119529666735
		pshRNA + MBs + US	0.849046377460902*	0.175638001537875	0.000	0.482671926302083	1.215420828619720
	pshRNA + US	sham	−0.333818251722829	0.175638001537875	0.072	−0.700192702881648	.032556199435989
		NS	−1.630215263113970*	0.175638001537875	0.000	−1.996589714272788	−1.263840811955151
		pshRNA	−0.532745078507917*	0.175638001537875	0.007	−0.899119529666735	−0.166370627349098
		pshRNA + MBs + US	0.316301298952985	0.175638001537875	0.087	−0.050073152205833	0.682675750111803
	pshRNA + MBs + US	sham	−0.650119550675814*	0.175638001537875	0.001	−1.016494001834633	−0.283745099516996
		NS	−1.946516562066955*	0.175638001537875	0.000	−2.312891013225773	−1.580142110908136
		pshRNA	−0.849046377460902*	0.175638001537875	0.000	−1.215420828619720	−0.482671926302083
		pshRNA + US	−0.316301298952985	0.175638001537875	0.087	−0.682675750111803	.050073152205833
Tamhane	Sham	NS	−1.296397011391140*	0.211535545768347	0.035	−2.472988043378227	−0.119805979404054
		pshRNA	−0.198926826785087	0.116628538747879	0.832	−0.847631479547262	0.449777825977087
		pshRNA + MBs + US	0.333818251722829	0.115059613885239	0.363	−0.306159816027175	0.973796319472834
		NS	0.650119550675814*	0.074387842011638	0.009	0.236363708924220	1.063875392427408
	NS	sham	1.296397011391140*	0.211535545768347	0.035	0.119805979404054	2.472988043378227
		pshRNA	1.097470184606053*	0.241556418200755	0.035	0.077947592677843	2.116992776534263
		pshRNA + US	1.630215263113970*	0.240802827788489	0.004	0.610042140263328	2.650388385964612
		pshRNA + MBs + US	1.946516562066955*	0.224233891645890	0.003	0.878613164315598	3.014419959818311

(*Continued*)

Table S2: *Continued*

Multiple comparisons						
**(I) 1**	**(J) 1**	**Average difference (I-J)**	**standard error**	**significance**	**95% Confidence interval**	
					**lower limits**	**upper limits**
pshRNA	sham	0.198926826785087	0.116628538747879	0.832	−0.449777825977087	0.847631479547262
	NS	−1.097470184606053*	0.241556418200755	0.035	−2.116992776534263	−0.077947592677843
	pshRNA + US	0.532745078507917	0.163832020062886	0.111	−0.092534914941883	1.158025071957717
	pshRNA + MBs + US	0.849046377460902*	0.138332089876550	0.005	0.287245091552980	1.410847663368824
pshRNA + US	sham	−0.333818251722829	0.115059613885239	0.363	−0.973796319472834	0.306159816027175
	NS	−1.630215263113970*	0.240802827788489	0.004	−2.650388385964612	−0.610042140263328
	pshRNA	−0.532745078507917	0.163832020062886	0.111	−1.158025071957717	0.092534914941883
	pshRNA + MBs + US	0.316301298952985	0.137011918410658	0.433	−0.238298136270600	0.870900734176570
pshRNA + MBs + US	sham	−0.650119550675814*	0.074387842011638	0.009	−1.063875392427408	−0.236363708924220
	NS	−1.946516562066955*	0.224233891645890	0.003	−3.014419959818311	−0.878613164315598
	pshRNA	−0.849046377460902*	0.138332089876550	0.005	−1.410847663368824	−0.287245091552980
	pshRNA + US	−0.316301298952985	0.137011918410658	0.433	−0.870900734176570	0.238298136270600

Homogeneous subset					
**1**					
	**1**	***N***	**A subset of alpha = 0.05**		
			**1**	**2**	**3**
Student-Newman-Keuls multiple comparisons	pshRNA + MBs + US	5	0.349880449324186		
	pshRNA + US	5	0.666181748277171	0.666181748277171	
	sham	5		1.000000000000000	1.000000000000000
	pshRNA	5			1.198926826785087
	NS	5			
	significance		0.087	0.072	0.271

(*Continued*)

Table S2: *Continued*

	**1**	
	**1**	**A subset of alpha = 0.05**
		**4**
Student-Newman-Keuls multiple comparisons [a]	pshRNA + MBs + US	
	pshRNA + US	
	sham	
	pshRNA	
	NS	2.296397011391140
	significance	1.000

*. Differences in means are significant at the 0.05 level.

The average value of the group in a homogeneous subset is displayed. [a]Use the harmonic mean sample size = 5.000.
